# The Effect of Periodic Email Prompts on Participant Engagement With a Behavior Change mHealth App: Longitudinal Study

**DOI:** 10.2196/43033

**Published:** 2023-05-11

**Authors:** Elena Agachi, Tammo H A Bijmolt, Koert van Ittersum, Jochen O Mierau

**Affiliations:** 1 Department of Marketing Faculty of Economics and Business University of Groningen Groningen Netherlands; 2 Department of Economics, Econometrics & Finance Faculty of Economics and Business University of Groningen Groningen Netherlands

**Keywords:** mobile health, behavior change, mobile app, digital health, engagement, retention, email, hidden Markov model

## Abstract

**Background:**

Following the need for the prevention of noncommunicable diseases, mobile health (mHealth) apps are increasingly used for promoting lifestyle behavior changes. Although mHealth apps have the potential to reach all population segments, providing accessible and personalized services, their effectiveness is often limited by low participant engagement and high attrition rates.

**Objective:**

This study concerns a large-scale, open-access mHealth app, based in the Netherlands, focused on improving the lifestyle behaviors of its participants. The study examines whether periodic email prompts increased participant engagement with the mHealth app and how this effect evolved over time. Points gained from the activities in the app were used as an objective measure of participant engagement with the program. The activities considered were physical workouts tracked through the mHealth app and interactions with the web-based coach.

**Methods:**

The data analyzed covered 22,797 unique participants over a period of 78 weeks. A hidden Markov model (HMM) was used for disentangling the overtime effects of periodic email prompts on participant engagement with the mHealth app. The HMM accounted for transitions between latent activity states, which generated the observed measure of points received in a week.

**Results:**

The HMM indicated that, on average, 70% (15,958/22,797) of the participants were in the inactivity state, gaining 0 points in total per week; 18% (4103/22,797) of the participants were in the average activity state, gaining 27 points per week; and 12% (2736/22,797) of the participants were in the high activity state, gaining 182 points per week. Receiving and opening a generic email was associated with a 3 percentage point increase in the likelihood of becoming active in that week, compared with the weeks when no email was received. Examining detailed email categories revealed that the participants were more likely to increase their activity level following emails that were in line with the program’s goal, such as emails regarding health campaigns, while being resistant to emails that deviated from the program’s goal, such as emails regarding special deals.

**Conclusions:**

Participant engagement with a behavior change mHealth app can be positively influenced by email prompts, albeit to a limited extent. Given the relatively low costs associated with emails and the high population reach that mHealth apps can achieve, such instruments can be a cost-effective means of increasing participant engagement in the stride toward improving program effectiveness.

## Introduction

### Background

Following the increasing need for the prevention of noncommunicable diseases [1], behavior change programs have emerged as a widely used support tool for health interventions aimed at improving lifestyle behaviors [2]. Digital behavior change programs have the ability to reach a larger population subset at relatively lower costs than their offline counterparts [3,4] while allowing for tailored material based on individual interactions [5].

In recent years, mobile health (mHealth) apps have gained traction as an increasingly preferred method of delivering digital behavior change interventions [4] by further facilitating access for and interaction with participants [6]. An additional benefit of mHealth apps is their ability to also involve the population segment with lower socioeconomic conditions, which generally shows less interest in preventive health interventions [7-9].

Although digital behavior change programs, especially mHealth apps, are promising tools for improving lifestyle behaviors, in practice, these programs often show low participant engagement (defined as “the extent of usage of the digital behavior change intervention” [10]) and high attrition rates [11-13]. One of the main reasons underlying this phenomenon is the passive nature of behavior change programs, where participants need to act by themselves to benefit [14]. Although higher engagement is crucial for achieving effectiveness of mHealth apps [15-18], inducing higher participant engagement over time is a challenging task [12,19], which requires proactive efforts from the program providers [11,20,21].

### Objective

Periodic prompts via emails have been examined as a potential tool that can boost participant engagement with behavior change mHealth apps [22,23]. However, most studies examining the means to increase engagement with an mHealth app are based on small sample sizes and short time spans [24,25]. In a literature review including approximately 35 mHealth apps aimed at increasing physical activity, the sample size varied between 8 and 700 participants, with an average study duration of 8 weeks [26]. Given that small sample sizes and especially short time spans of most interventions can lead to an overestimation of the intervention effects [25,27], it is essential to examine whether periodic prompts via emails can impact participant engagement with a behavior change mHealth app within a longer-term, larger-scale, noncontrolled setup [7,28,29].

This study relied on a large-scale (more than 20,000 participants), open-access mHealth app focused on improving the lifestyle behaviors and wellness of its participants. The analysis in this study used a hidden Markov model (HMM) to examine whether periodic email prompts were able to increase participant engagement with the mHealth app and how this effect evolved over time. By investigating the effect of prompts on continued engagement with the mHealth app, this study hoped to assess (1) whether periodic prompts via email can be a viable tool for increasing participant engagement, (2) how the impact of periodic prompts on engagement evolved over time, and (3) how the observed effects differed among participant subgroups.

## Methods

### Study Sample

This study was based on data from the mobile app of a digital behavior change program operated in the Netherlands. The program’s goal was to improve the wellness and lifestyle behaviors of its participants by promoting physical activity, healthy eating habits, social activity, mental health, good sleep habits, and minimized stress. The mobile app was introduced in October 2017, providing functions such as entering or recording physical activities, reading articles, setting goals, including friends in challenges, answering health questions, being assisted by a web-based coach, and forming a daily “fit-score.” On the basis of the individual activities in the mobile app, participants gain points, which can be used to acquire specific products, vouchers for various services, or make charity contributions.

The data analyzed in this study spanned from January 2018, when the mobile app of the health program reached full functionality, to July 2019, when the observation window ended. Data were collected in 2018 and 2019 and analyzed in 2021 and 2022 within a longitudinal, nonexperimental study design. The analyzed data had a weekly frequency, covering 78 weeks and including 22,797 unique participants who enrolled by themselves in the mobile app at any time during the observation window. Of the 33,825 participants who used the mobile app, 22,797 (67.4%) were included, having at least 1 activity during the period between the mobile app introduction and the end of the observation window, indicating an awareness of the mobile app’s functionality. All program participants were aged between 18 and 80 years and were residents of the Netherlands.

Enrollment in the mHealth app was open and free, and all the participants involved in this study provided their voluntary and informed consent.

### Ethics Approval

Ethics approval for this research project based on the health program was obtained from the institutional research board of the University of Groningen (approval number RDMPFEB20180831-7309).

### Measures

#### Participant Engagement

The main objective of a behavior change mHealth app is to improve the lifestyle behaviors of its participants. Achieving effectiveness in behavior change is highly linked to the degree to which participants engage with the app; only when participants interact with the program and continue use can it have an impact on their behavior [15,16].

Participant engagement has been defined as “the extent of usage of the digital behavior change intervention” [10], being separated into temporal patterns—frequency and duration, and depth—specific intervention content use [30,31]. Participant engagement can be assessed as a subjective measure (ie, self-reported by participants) or an objective measure (ie, measured by the program) [10].

The mHealth app analyzed in this study provided several activities that the participants could perform. For every activity completed, the participants gained points, which varied based on the activity type and duration. Gained points were used as a measure of the activity level of the participants in the mHealth app, which were calculated weekly throughout the observation window. Consequently, this participant engagement measure was objective rather than self-reported, which had the additional benefit of being more robust against reporting bias [32].

The 2 types of activities included in the participant engagement measure were physical activities and web-based coach activities, both of which the participants could access via the mHealth app. Physical activities were activities recorded in the health program with the use of GPS, such as walking, cycling, and running. Web-based coach activities were the interactions that the participants had with regular messages sent via the chat environment programmed by the providers of the mHealth app. The messages in the chat environment were linked to physical activities, health goals that the participants selected, challenges that they joined, or overall health behavior information. For every question answered, the participants received a fixed number of points. Table 1 presents the activity types included in the mHealth app, with the associated number of points.

**Table 1:** Activity types and number of points gained.

Activity type	Number of points
Web-based coach	1 point gained for any question answered
GPS recorded activity: walking	From 1 point to 696 points, depending on activity duration (mean 17.25, SD 24.46 points)
GPS recorded activity: cycling	From 1 point to 699 points, depending on activity duration (mean 21.37, SD 38.36 points)
GPS recorded activity: running	From 1 point to 695 points, depending on activity duration (mean 39.93, SD 33.06 points)

#### Periodic Email Prompts

In general, mHealth apps suffer from low participant engagement and high dropout rates [33,34]. Capturing the attention of the participants in an attempt to stimulate their active involvement is crucial for program success [20]. Periodic email prompts are often used as tools for improving participant engagement, with mixed results. Although some studies estimated a positive impact of email prompts on participant engagement [23,28,35], additional research is required in this area [36], with a special focus on overtime effects [10,22].

In this study, we examined the ability of periodic email prompts to improve participant engagement with the mHealth app by measuring the effect of emails on transitions between activity states. For every email sent, a randomly selected subset of the participants did not receive the email in question, which served as the control group for that particular email. The emails sent to the participants of the app were either generic or targeted. The generic emails were sent to all the participants in the same format, independent of their current activity level. The targeted emails were sent out in different versions, depending on the participants’ activity level (low activity or high activity); however, it was not possible to identify which participant received which email version. The targeted emails could belong to one of the following categories: welcoming emails, reactivation emails, recruitment emails, newsletters, health campaigns, and special offers.

To correct for any potential effect of targeting, generic emails were the main measure used in this study. The emails included in this category had topics such as welcoming participants to the program, sharing general healthy lifestyle information (at regular intervals), presenting topic-specific health information (eg, healthy nutrition and sleep), inviting participants to engage in activities, and presenting special deals in the web shop.

To measure the effect of email prompts, we distinguished between 3 situations: not having received an email, having received an email but not having opened it, and having received an email and opened it. Section 2 in Multimedia Appendix 1 describes in detail the emails’ content and their categorization.

Between January 2018 and July 2019, the proportion of participants who received a generic email varied between 0.14% (13/9596 participants in the third week of March 2018) and 39.73% (8943/22,508 participants in the second week of May 2019). The proportion of participants who opened the generic email when they received it varied between 26.65% (271/1017 participants in the third week of November 2018) and 76.1% (159/209 participants in the first week of January 2018). Across the weeks in the observation period, the average proportion of participants who received a generic email in a week was 6.6%, the average proportion of participants who opened a generic email in a week was 3.5%, and the average proportion of participants who opened a generic email when they received one was 60.5%.

**Figure 1:**
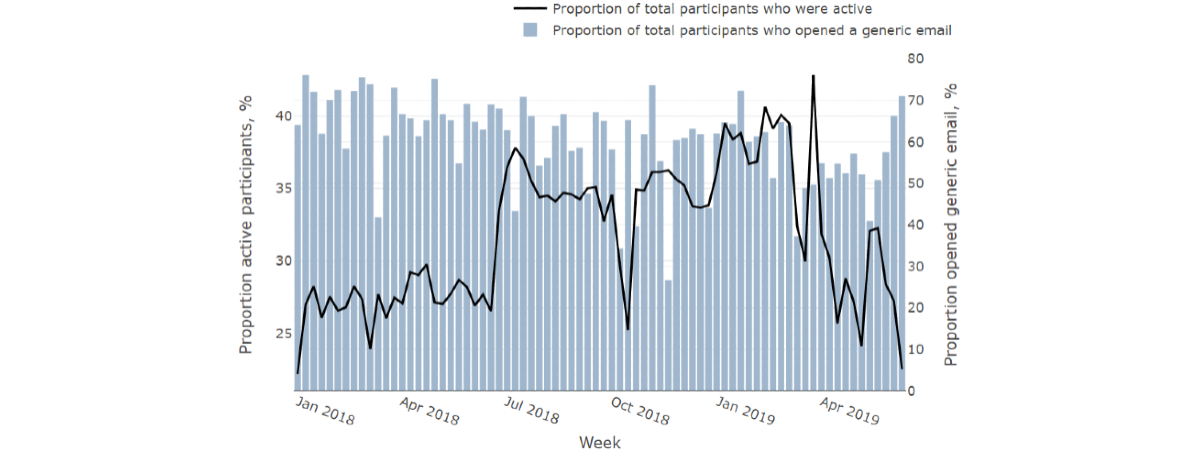
The proportion of active participants (at least 1 point gained) and the proportion of participants who received and opened a generic email.

Figure 1 displays the evolution of the generic emails and participant engagement over the weeks of the observation window, showing the proportion of participants who received and opened a generic email and the proportion of participants who were active during that week (having gained at least 1 point). Figure 1 implies great variability in both measures, highlighting the need to analyze the connection between email prompts and participant engagement in a dynamic manner.

To control for the effect of the individual characteristics of the program participants on their engagement with the behavior change mHealth app [10], gender, age, and neighborhood socioeconomic status (NSES) [37] quintiles were included in the analysis as additional covariates. The NSES quintiles measure follows the methodology outlined in Dekker et al [38], being calculated using nonlinear iterative partial least squares principal component analysis on the following characteristics given on a postcode level: average income, average property value, subsidized renting, share of high-income households, share of owner-occupied properties, share of low-income households, share of population receiving unemployment benefits, share of people receiving disability benefits, and share of people receiving short-term unemployment benefits. A lower NSES quintile corresponds to lower levels of socioeconomic conditions. In addition, to measure whether early adopters of the mHealth app showed higher engagement [39], we included the additional measure of early adoption, which corresponded to the participants who enrolled in the behavior change mHealth app during its first month of existence. In total, 19.06% (4345/22,797) of the mHealth app participants were early adopters.

### Statistical Analysis

In this study, we used the number of points gained per week to measure engagement, which reflected the level of activity of a participant in the mHealth app. To model the changing levels of activity over time, an HMM was used, where a participant had a specific level of activity each week (latent state) and could transition between the activity states from week to week [40,41]. Using HMMs allows for the disentanglement of the dynamics of participant behavior over time and the analysis of how specific actions can influence these behaviors [42]. Moreover, the HMM model is preferred because such a latent approach allows for the incorporation of the high dropout and inactivity rates that are specific to behavior change programs [43,44]. To understand the drivers of the dynamics of state transitions, nonhomogeneous Markov modeling was used, which allows the transition probabilities to depend on time-varying covariates [45].

**Figure 2:**
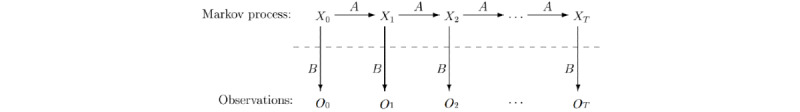
A generic hidden Markov model representation. A: state transition probability; B: response probability matrix; O: observations in the response vector; T: last measurement week.

A generic HMM is defined as shown in Figure 2, where *X_t_* is the latent activity state at time *t,* with *t* ranging from *0* to *T* (*T* being the last measurement week); *A* is the state transition probability; *B* is the response probability matrix; and *O_t_* contains the observations in the response vector. The Markov process, being separated by the dashed line, was not observed. Instead, only the observations *O_t_* were known; in this study, these were the number of points gained in a week.

The HMM depicted in Figure 2 consists of 3 main elements (as shown below):

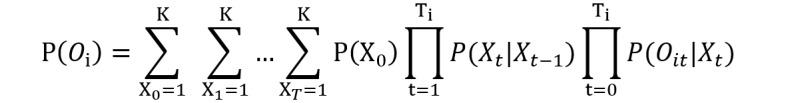



The hidden Markov model formulation includes:

1. Initial state probability *P(X0)*: the probability that participant *i* is in state *X* at time 0.
2. Transition probability *P(Xt|Xt-1)*: the probability that participant *i* is in state *X* at time *t*, given the state membership at time *t-1*.
3. Response probability *P(Oit|Xt)*: the probability that participant *i* displays activity level *O* at time *t*, given the state membership *X* at time *t*.

In the setup of this study, the unobserved states that a participant belonged to were activity states, generating the observed measures of points received in a week from differing activities performed in the mHealth app. The initial state distribution reflected the starting state that a participant belonged to at their moment of joining the mHealth app, which depended on the time-constant covariates that reflected the participant’s background (age, gender, NSES, and early adopter). The transitions between the activity states reflected the variability in the participants’ behaviors between weeks, which were allowed to depend on both time-constant covariates (age, gender, NSES, and early adopter) and time-varying covariates (the email prompts and time). Including the email prompts in the transition probability model allowed for the examination of the impact of emails on changes in the activity levels of a participant.

When using the HMM for general inference, traditional model selection criteria, such as Akaike information criterion or Bayesian information criterion, often lead to the selection of much larger numbers of states than expected a priori [46-48]. The reason for this is that the neglected data in the model formulation are absorbed into the additional model states, which do not possess a clear interpretation anymore [49]. A recommended approach for dealing with this uncertainty is analyzing a prespecified number of latent states. In this study, following the goal of differentiating between activity states while prioritizing interpretability, the estimated HMM contained 3 states: inactivity, average activity, and high activity. For the estimation of the model, the Latent GOLD software (Statistical Innovations Inc) was used. The Latent GOLD software supports the analysis of latent class models such as HMMs, with the parameter estimates being computed based on a combination of expectation-maximization and Newton-Raphson iterations, where the E step computations use a forward-backward recursion scheme [50].

## Results

### Characteristics of the Participants

This study analyzed 22,797 participants between January 2018 and July 2019 for a total of 78 weeks. The mHealth app analyzed being an open-access platform, the participants could enroll at any time within the observation window. Table 2 outlines the characteristics of the mHealth app participants. On average, each participant was observed for 50.8 weeks, resulting in a total of 1,129,706 observation points. Every week, approximately one-third of the study population was active, gaining an average of 28 points weekly. A total of 62.82% (14,321/22,797) of the analyzed participants were women, with the most represented age group being between 37 and 46 years and the highest proportion of participants belonging to the second socioeconomic quintile.

**Table 2:** Characteristics of the study participants (N=22,797).

Key attributes			Values
Total observations, n			1,129,706
Participants, n			22,797
Number of weeks in the mobile app, mean (SD)			50.8 (28.7)
Number of points received weekly, mean (SD)			28.0 (78.8)
Proportion of active participants per week (%), mean (SD)			31.1 (6.3)
Early mobile app adopters, n (%)			4345 (19.06)
Female participants, n (%)			14,321 (62.82)
**Participants per age group (years), n (%)**			
	18-26	1408 (6.18)	
	27-36	5282 (23.17)	
	37-46	5406 (23.71)	
	47-56	5179 (22.72)	
	57-66	3402 (14.92)	
	67-80	2120 (9.3)	
**Participants per NSES^a^ quintile (from the lowest to the highest socioeconomic conditions), n (%)**			
	First	4675 (20.51)	
	Second	6303 (27.65)	
	Third	4662 (20.45)	
	Fourth	3611 (15.84)	
	Fifth	3546 (15.55)	

^a^NSES: neighborhood socioeconomic status.

### Model Estimation Results

#### HMM States and Transitions

Estimating the HMM with 3 states resulted in the outcomes presented in Tables 3 and 4. The 3 states identified by the HMM were labeled as the inactivity state, average activity state, and high activity state. On average, across the weeks of the observation period, 70% (15,958/22,797) of the participants were in the inactivity state, gaining 0 points weekly; 18% (4103/22,797) of the participants were in the average activity state, gaining 27 points weekly; and 12% (2736/22,797) of the participants were in the high activity state, gaining 182 points weekly.

**Table 3:** Hidden Markov model estimation results: average latent states.

	State		
	1: inactivity	2: average activity	3: high activity
Average state size (%)	70	18	12
Points received, n	0	27	182

**Table 4:** Hidden Markov model estimation results: average transition probability matrix^a^.

State (t-1)	State (t^b^)		
	1	2	3
1	0.91	0.07	0.02
2	0.31	0.64	0.05
3	0.09	0.09	0.82

^a^Section 3 in Multimedia Appendix 1 presents detailed model fit criteria and parameter estimates for the hidden Markov model. All the parameter estimates were statistically significant at the 99% confidence level.

^b^t: time point.

The estimated transition matrix (shown in Table 4) reflects the probability of switching between the 3 states across weeks. On average, the inactivity and high activity states were most persistent, for example, a participant who was in the high activity state during week 1 was, on average, 82% likely to remain in that state during week 2. The highest probability of decrease in activity was associated with the transition from the average activity state to the inactivity state: a participant who was in the average activity state during week 1 was 31% likely to transition into the inactivity state during week 2.

#### HMM Effects of Generic Email Prompts

The relationship of interest in this study is the connection between generic emails and participant engagement. Table 5 shows the estimated posterior probability means of the state distribution depending on whether the participants received and opened a generic email. The posterior probability means indicate the estimated probability that a participant was in each state, given the email prompt. The estimates show that a participant who did not receive a generic email was 68% likely to be in the inactivity state, whereas a participant who received and opened a generic email was 67% likely to be in the inactivity state (a decrease of 1 percentage point). In addition, the participants who received but did not open a generic email were estimated to have a 11 percentage point higher likelihood of inactivity than those who did not receive an email.

**Table 5:** Hidden Markov model estimation results: posterior probability means associated with the generic email prompts^a^.

	1: inactivity	2: average activity	3: high activity
No generic email received	0.68	0.19	0.13
Generic email received but not opened	0.79	0.14	0.06
Generic email received and opened	0.67	0.22	0.11

^a^Section 3 in Multimedia Appendix 1 presents detailed model fit criteria and parameter estimates for the hidden Markov model. All the parameter estimates were statistically significant at the 99% confidence level.

As the HMM allowed for the dynamics of switching between states, Table 6 shows the estimated transition matrices depending on the generic email. On the basis of the estimated transition matrices, the likelihood that a participant remained in the inactivity state between weeks *t* and *t+1* was 91% when no email was received, as opposed to 88% when a generic email was received and opened. This translates into a 3 percentage point decrease in the probability of remaining inactive or, alternatively, a 3 percentage point increase in the probability of moving into one of the activity states after receiving and opening a generic email.

**Table 6:** Hidden Markov model estimation results: transition matrices accounting for generic email prompts.

State (t^a^-1)	State (t)										
	Transition matrix: no generic email received				Transition matrix: generic email received but not opened				Transition matrix: generic email received and opened		
	1	2	3	1		2	3	1		2	3
1	0.91	0.07	0.02	0.93		0.06	0.01	0.88		0.10	0.02
2	0.31	0.64	0.05	0.41		0.56	0.03	0.29		0.66	0.04
3	0.09	0.09	0.82	0.11		0.13	0.76	0.08		0.10	0.82

^a^t: time point.

#### HMM Effects of Detailed Email Prompts

To examine whether the effect of the email prompts differed based on email type, the estimated posterior probability means of the state distribution was formulated depending on whether the participants received and opened an email using detailed email categories (Table 7). On the basis of the posterior probability means shown in Table 7, both positive and negative effects could be identified, where a positive effect reflects an increase in participant activity linked to receiving and opening an email, whereas a negative effect reflects the opposite. A positive effect was associated with opening a welcome email (decreased likelihood of inactivity by 12 percentage points) and opening a health campaign email (decreased likelihood of inactivity by 3 percentage points). A negative effect was associated with opening a newsletter or special offer email (increased likelihood of inactivity by 2 percentage points) and opening a reactivation email (increased likelihood of inactivity by 5 percentage points).

**Table 7:** Hidden Markov model estimation results: posterior probability means accounting for detailed email prompts^a^.

			1: inactivity		2: average activity		3: high activity
No email received			0.68		0.19		0.13
**Welcome email**							
	Email received but not opened	0.70		0.24		0.06	
	Email received and opened	0.56		0.34		0.10	
**Reactivation email**							
	Email received but not opened	0.85		0.11		0.04	
	Email received and opened	0.73		0.18		0.09	
**Newsletter email**							
	Email received but not opened	0.80		0.13		0.07	
	Email received and opened	0.70		0.18		0.12	
**Health campaign email**							
	Email received but not opened	0.75		0.19		0.06	
	Email received and opened	0.65		0.26		0.09	
**Special offer email**							
	Email received but not opened	0.83		0.12		0.05	
	Email received and opened	0.70		0.19		0.11	

^a^Section 3 in Multimedia Appendix 1 presents detailed model fit criteria and parameter estimates for the hidden Markov model. All parameter estimates were statistically significant at the 95% confidence level.

#### HMM Effects of Time and Background Characteristics

Examining the impact of generic email prompts on participant engagement over time revealed the estimated transition matrices and posterior probability means provided in section 4 in Multimedia Appendix 1. During the first half year, 72% (16,413/22,797) of the participants were estimated to be in the inactivity state, which decreased to 65% (14,818/22,797) during the second half year. For the last half year observed, 69% (15,730/22,797) of the participants were estimated to be in the inactivity state. The impact of receiving and opening a generic email on the transition probabilities did not change much over time, being associated with a decreased likelihood of remaining in the inactivity state by 2 percentage points in the first and third half years and 3 percentage points in the second half year.

The background characteristics of the participants were also linked to differences in activity levels. On the basis of the estimated HMM model (transition matrices and posterior probability means shown in section 5 in Multimedia Appendix 1), female participants were more likely to be in the inactivity state and less likely to be in the high activity state compared with male participants (with a difference of 6 percentage points). The lowest socioeconomic group and the youngest age group were associated with a higher likelihood of inactivity, whereas the age group from 47 to 56 years was the most active. Finally, being an early mHealth app adopter was associated with a 5 percentage point decrease in the likelihood of being in the inactivity state (with the same level of increase in the likelihood of being in the high activity state).

The impact of the generic email prompts on participant engagement did not vary substantially between participants depending on their age, gender, or NSES quintile, with the only difference being that the participants in the oldest age group (67 to 80 years) had a higher likelihood of transitioning toward one of the activity states after opening a generic email than the other age groups (an effect of 4 percentage points).

To examine the robustness of the above-discussed results, several additional models were estimated, with the results confirming those presented in this study. Section 6 in Multimedia Appendix 1 contains several alternative specifications of the HMM model and their estimation results, namely using an indicator for any email received (independent of the email type or targeting nature) and incorporating the email prompts as covariates in the response probabilities model. In addition, following the Strengthening the Reporting of Observational Studies in Epidemiology (STROBE) recommendations, the checklist presented in Multimedia Appendix 2 was completed.

## Discussion

### Principal Findings

Digital behavior change programs are widely implemented as a means of improving lifestyle behaviors and population health. However, such programs often exhibit low participant engagement rates, with additional effort needed from the program providers to stimulate and maintain engagement. This study analyzed the ability of email prompts to increase participant engagement with an mHealth app aimed at supporting behavior change. Although email prompts showed some positive results in stimulating engagement, their effect in a large-scale, nonexperimental setting with a longer time span is still unclear.

The analysis in this study used an HMM to disentangle the dynamics around email prompts and participant engagement. The estimated HMM with 3 latent states revealed that, on average, 70% (15,958/22,797) of the participants were in the inactivity state, gaining 0 points weekly; 18% (4103/22,797) of the participants were in the average activity state—gaining 27 points weekly; and 12% (2736/22,797) of the participants were in the high activity state—gaining 182 points weekly.

Focusing on the effect of generic emails, the estimation results indicated that when allowing for time dependency, receiving and opening a generic email was associated with a 3 percentage point lower likelihood of remaining inactive than when no email was received (equivalent to a 3 percentage point increase in the likelihood of transitioning from inactivity to one of the activity states). By contrast, receiving but not opening a generic email was associated with a higher likelihood of inactivity. This observed negative impact that receiving but not opening an email had on participant engagement can be explained by the higher proportion of inactive participants in the group that did not open the email than in the group that did not receive an email. Given that the average opening rate of the emails sent within the analyzed mHealth app was above 60%, it can be argued that some increase in participant engagement is possible with the use of generic emails; however, additional effort should be directed at ensuring high opening rates for the emails sent.

Allowing the effect of generic emails to vary over time revealed a relatively stable pattern: in all 3 half-year periods analyzed, the likelihood of moving out of inactivity after opening a generic email was between 2 and 3 percentage points, with the strongest effect corresponding to the second half year of the observation period. Estimating the association of the effects observed with participant background characteristics showed that being a male, being older, having a higher socioeconomic status, and being an early adopter of the mHealth app were all factors associated with higher participant engagement. The impact of the generic emails did not significantly depend on the participants’ age, gender, or NSES quintile, with the only difference being that the participants in the age group of 67 to 80 years were more likely to move out of inactivity after receiving and opening a generic email than those in the other age groups.

This study further analyzed the impact of detailed email categories on participant engagement, revealing both negative and positive effects. On one hand, it was estimated that emails welcoming participants to the program and emails containing health-related information were associated with an increase in activity levels (12 and 3 percentage points, respectively). On the other hand, emails that contained generic program information, promoted special offers on products in the web shop, or were aimed at reactivating inactive users were linked to a decrease in activity levels (2, 2, and 5 percentage points, respectively). These findings imply that participants were more reactive to health-related information, which was in line with the program’s goals and potentially with the participants’ motivation for using the mHealth app, while being resistant to emails that deviated from the program’s goal of improving health behaviors. Moreover, the negative effect of the reactivation email implies that it is difficult to stimulate activity in participants who have been inactive for long periods, highlighting the importance of focusing on preventing participants from becoming inactive.

### Comparison With Previous Work

Digital behavior change programs often exhibit low participant engagement [11-13]. On the basis of a systematic review, Kelders et al [11] estimated that the average adherence to web-based lifestyle interventions is 23%, which is similar to the 30% of active participants identified in the mHealth app in this study. The slightly higher proportion of active participants estimated here can be because of the mobile app format of the behavior change intervention, which is associated with higher flexibility of use and more personalized content [51].

In an attempt to identify means of improving participant engagement, email prompts have been examined in the context of behavior change programs, with studies reporting small to moderate effects [23]. However, the effects of email prompts on participant engagement are often analyzed over a short period [25,27], subsequently diminishing [12,52] or even disappearing [25]. This study estimated that participant engagement increased by approximately 3 percentage points when an email was received and opened. A similar impact was seen in the study of Ryan et al [52], who based on average activity levels, observed an increase of approximately 3% in the steps taken on the days on which an email was sent. A possible reason for the limited effect of email prompts on engagement is that participants can find such reminders annoying [53]. Alternatively, in the case of targeted emails, inadequate personalization is another factor linked to low participant engagement [54]. Finally, inducing higher participant engagement over time is a challenging task [12,19], partially because of the passive nature of behavior change programs, where participants need to act by themselves to benefit [14].

Participant background characteristics are linked to their engagement with the mHealth app [55]. Similar to the findings in this study, previous work has also shown that males [52,56], older age groups [10,56,57], higher socioeconomic status participants [10,52], and early adopters [38] have higher engagement with mHealth apps. The observation that older age groups are more responsive to emails than other age groups can be explained by their appreciation of reminders within mHealth apps [58], indicating that such tools are especially efficient in increasing activity levels among the older population group.

The results of this study show that although email prompts can achieve a small to moderate increase in participant engagement, this tool alone is likely insufficient for increasing activity levels in an mHealth behavior change app. However, given that the costs associated with email prompts are relatively low, they may be a cost-effective means to improve participant engagement with an mHealth app when the program achieves a high population reach [7,59]. In addition, alternative program efforts, such as expert consultation or real-time feedback [7] could be used next to email prompts to further reduce participant dropout and improve the program effectiveness.

### Limitations and Future Research

There are several limitations around this study.

First, as a measure of participant engagement, this study solely used the activities recorded within the mHealth app. However, it is likely that the participants performed additional activities that were not recorded in the app environment. To overcome this limitation, one approach could be to combine the currently used objective measure of participant engagement with an additional subjective measure through which participants themselves can report their perceived activity level. Alternatively, a more accurate measure of activity could be achieved by extending the mHealth app to also include a wearable device, which could measure physical activity in a more precise manner.

Second, it is not a given that whenever a participant opens an email, they become aware of its content. It could be the case that some participants briefly open the email only to delete it, without reading any of its elements. In the setup of this study, we consider the action of opening the email to be a sufficient indication that the participant has been exposed to a reminder about the mHealth program analyzed. As a future extension to the current analysis, it could be of interest to examine whether reading the emails can lead to a higher impact on participant engagement. One possible way to measure whether participants read through the emails could be through the use of a clickable follow-up link, which can help distinguish between participants who pay attention to the content of the email and those who do not.

Third, the participants of the mHealth app analyzed in this study were older than 18 years. This excludes children and teenagers, with unclear insights into how the email prompts would work in increasing activity levels among these population groups. Given the importance of developing healthy lifestyle choices from a young age, a further extension to this study could be examining whether and how email prompts help increase activity among the younger population.

Finally, it is highly likely that the specific wording and topics addressed in an email have an impact on its effectiveness in increasing participant engagement. Although data on the detailed elements of the emails were not available in this study, analyzing such information could be a valuable extension. Namely, it would be of interest to examine how varying phrasing of the same topic and different levels of email personalization affect subsequent participant engagement.

### Conclusions

In this study, email prompts were examined as a tool for increasing participant engagement with a large-scale, open-access mHealth app with the goal of lifestyle behavior change. On the basis of an HMM allowing for weekly transitions between latent activity states, it was estimated that receiving and opening an email was associated with a small to moderate increase in participant engagement, which persisted over the 78 weeks analyzed. This finding suggests that email prompts can be used for improving participant engagement, albeit to a limited extent. However, given the relatively low costs associated with emails and the high population reach that mHealth apps can achieve, such instruments can be a cost-effective means of improving participant engagement to reduce dropout and improve the effectiveness of behavior change programs.

## Appendix

Multimedia Appendix 1 Web-based appendix with additional analysis information.

Multimedia Appendix 2 Strengthening the Reporting of Observational Studies in Epidemiology (STROBE) checklist.

## Abbreviations

HMM: hidden Markov model
mHealth: mobile health
NSES: neighborhood socioeconomic status
STROBE: Strengthening the Reporting of Observational Studies in Epidemiology
