# Supplementary material for: The Effect of Periodic Email Prompts on Participant Engagement With a Behavior Change mHealth App: Longitudinal Study
Source: JMIR Mhealth Uhealth. 2023 May 11;11:e43033. doi: 10.2196/43033 (PMC10214118; doi:10.2196/43033)
Supplement: Multimedia Appendix 1 [file mhealth_v11i1e43033_app1.docx]

# Appendix

# The Effect of Periodic Email Prompts on Participant Engagement with a Behavior Change mHealth App: Longitudinal Study

**Appendix Section 1: Representation of the virtual coach and the physical activity tracker**

Appendix Table 1. The virtual coach environment (left) and the physical activity tracker (right) inside the mHelath app


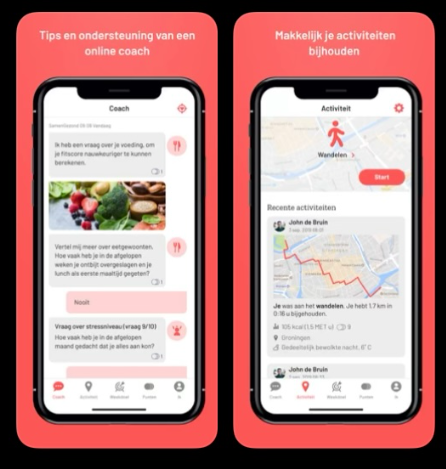


**Appendix Section 2: Email content and categorization**

Appendix Table 2. The emails prompts around the mHealh app and their content

| Email topic | Email content |
| --- | --- |
| welcome1 | Welcoming, immediately after enrollment |
| welcome2 | Additional information, 14 days after enrollment |
| welcome3 | Additional information, 28 days after enrollment |
| activ | Introduction and invitation to the mHealth app; sent to all participants, in 3 versions based on activity |
| friend | Inviting participants to introduce friends to the mHealth app; sent every week to active participants not contacted before |
| coins | Inviting participants to introduce co-insured to the mHealth app; sent every week to active participants not contacted before |
| day90nologin | Activating participants; sent after 90 days of no login; sent in two versions, for participants who earned points in the app and who did not |
| day180nologin | Activating participants; sent after 180 days of no login; sent in two versions, for participants who earned points in the app and who did not |
| year2nologin | Announcement about closing accounts due to EU regulations; sent to participants who did not login for two years, about pending account closure |
| quarter | Showing benefits of the mHealth app; sent in two versions, for active participants and not |
| quarterQ1,  Q2,Q3,Q4 | Quarterly emails with differing content; for example a product can be promoted, new features of the mHealth app can be highlighted, specific blogs can be promoted |
| targset | Inviting to set a saving goal, sent 28 days after enrollment, to participants who do not have a saving goal yet |
| targrem | Informing about a saving goal being removed from the webshop in 2 weeks; inviting to order product and set a new saving goal |
| setgoal | Inviting users to set a challenge in the mHealth app |
| nutri1 | Informing participants about healthy eating, allowing participants to ask questions to a dietician; sent in two versions, one to families, and one to risk groups |
| nutri2 sleep1,2,3 relax1,2,3 | Email campaigns with three parts; the first part is sent to all participants with information about the topic and a link for filling in a test on the topic; the second part is sent to a group with specific results on the test (for ex: to little overweight or not enough sleep groups); the third part is sent to all the respondents on the test, with a summary of the results |
| insurancesale | Informing users about change in insurance premium sale goal (nr. of points needed and number of times a year allowed to use); sent to users who have the insurance premium sale as a saving goal |
|  |  |
| deal, deal1, deal2, deal3 | Activating participants; sent to active participants; promoting supper deals and double points: show 2 products prized attractively for a short time period, and sometimes also promote "double points" for a week or a weekend |
| retent | Informing about the benefits of the mHealth app, sent to all participants who logged in, in 2 versions, to active participants and not |
| review | Asking participants for a review, 14 days after placing a webshop order |
| resol | Asking participants about their good intentions this year, sent in January |
| closure7day,  14day, 30day | Emails sent after year2nologin, at 7, 14, and 30 days before account closure |
| doactiv | Activating participants to engage in app activities, sent after 60,75 or 90 days after sign up |
| feedback | Asking information about opinion of the mHealth app, sent after  60, 75 or 90 days after sign up |
| blog | Several health blogs are promoted |
| order1 order2 | Activating participants to order products; sent after 60, 75 or 90 days after signing up |
| mile4 | Promoting the a running event in the north of the Netherlands (the program is a partner of the event) |
| bday | Sent on participant’s birthday, inviting user to login in and receive points, or choose present |

Appendix Table 3. The email types included in the generic email category

| Email category | Email topic |
| --- | --- |
| Generic | welcome1, welcome2, welcome3, quarterQ1, quarterQ2, quarterQ3, quarterQ4, blog, nutri1, sleep1, relax1, doactiv, targset, setgoal, resol, bday, deal, deal1, deal2, deal3 |

Appendix Table 4. The email types included in each detailed email category

| Email category | Email topic |
| --- | --- |
| Welcome | welcome1, welcome2, welcome3 |
| Reactivation | day90nologin, day180nologin, year2nologin, retent, closure7day, closure14day, closure30day,doactiv, order1, order2, targset, targrem, setgoal |
| Recruitment | friend, coins, activ |
| Newsletters | quarter, quarterQ1, quarterQ2, quarterQ3, quarterQ4, blog |
| Health campaigns | nutri1, nutri2, nutri3, sleep1, sleep2, sleep3, relax1, relax2, relax3 |
| Special offer | deal, deal1, deal2, deal3, mile4, insursale |

**Appendix Section 3: Model fit and parameter estimates for the HMM model**


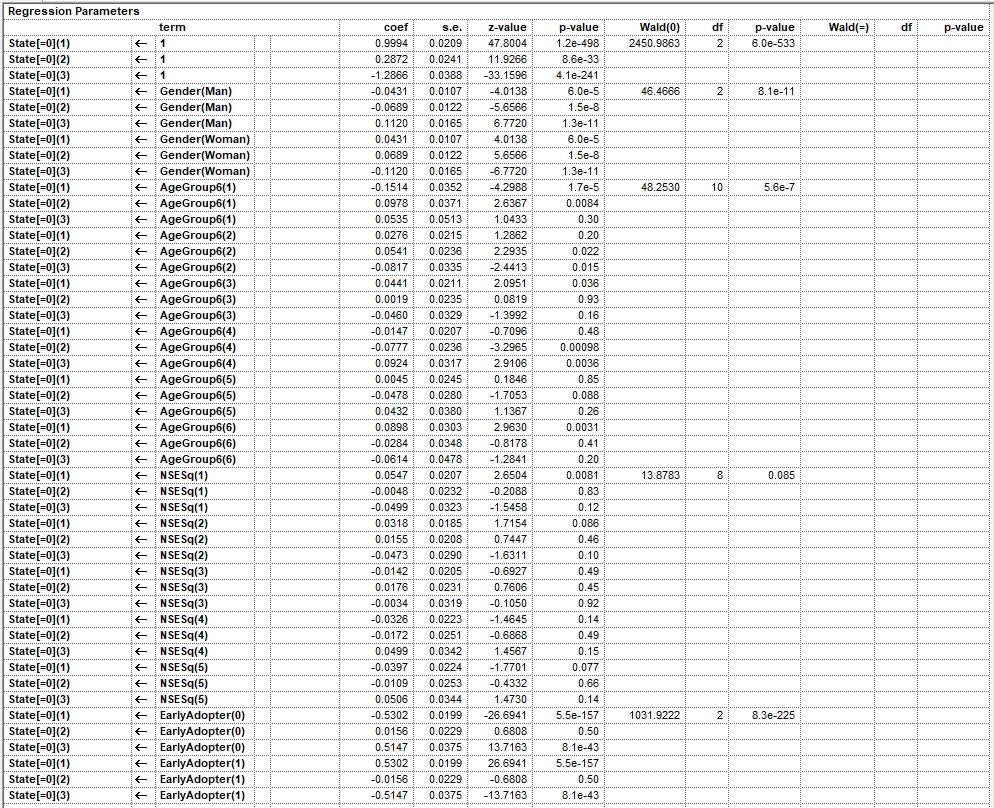


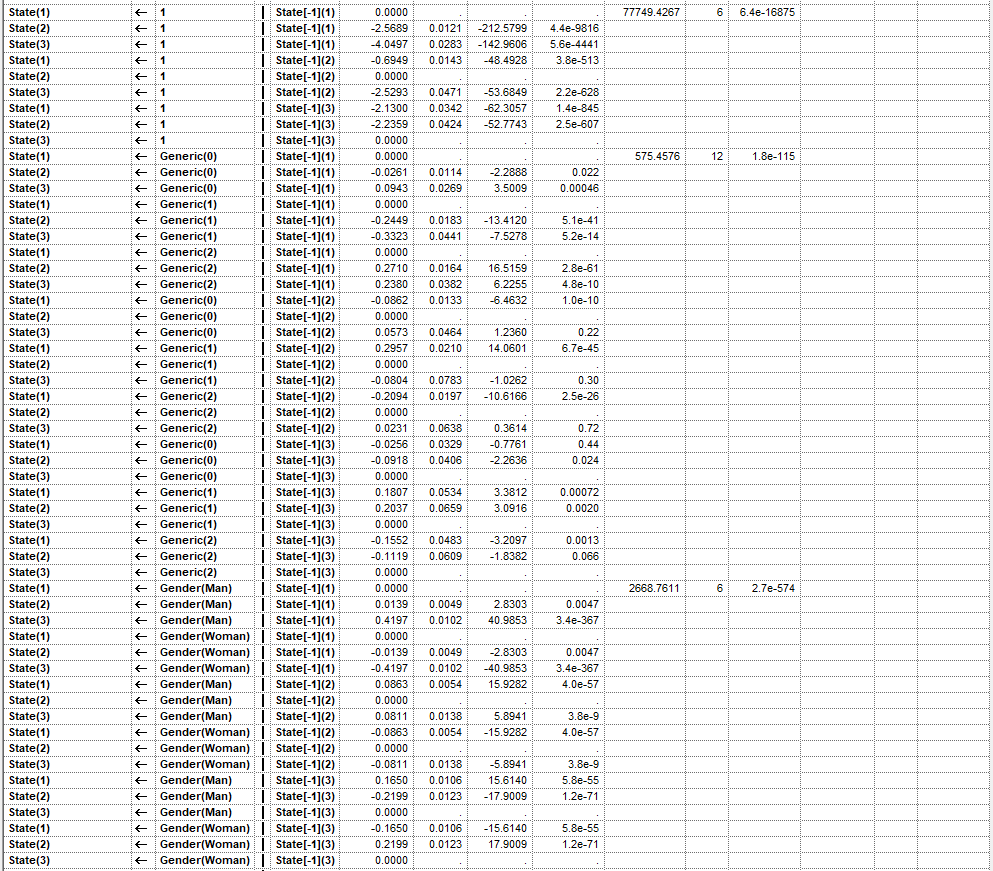


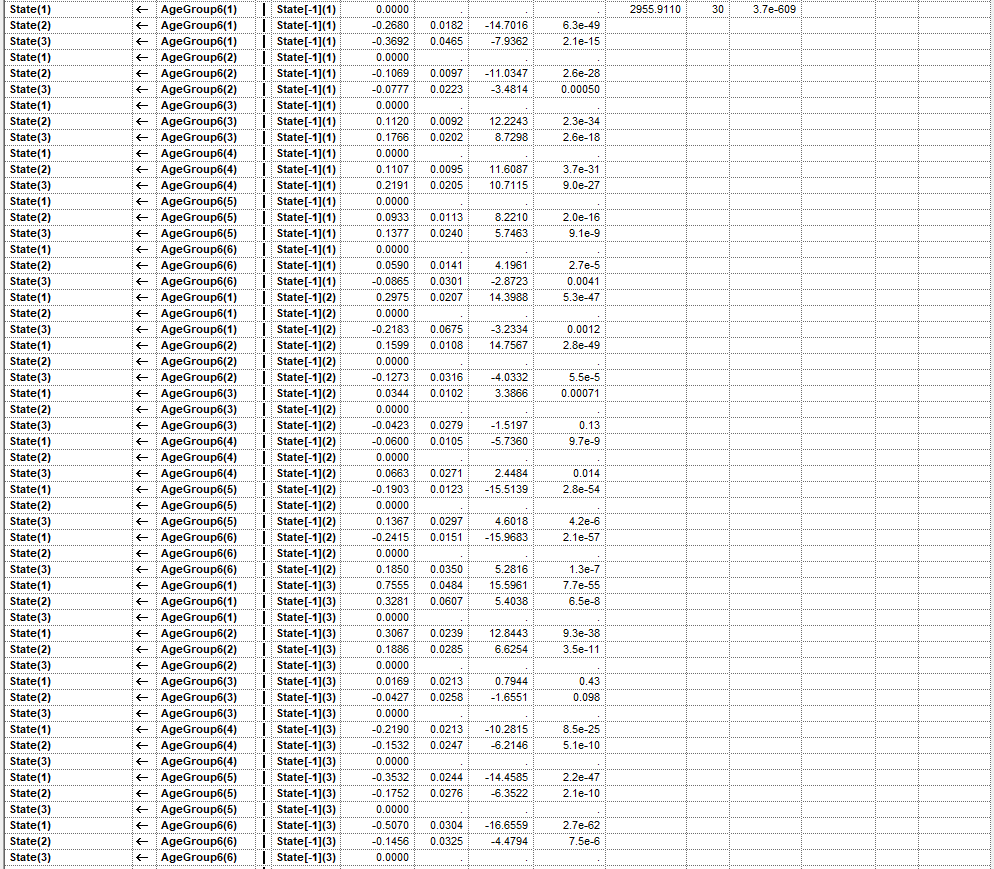


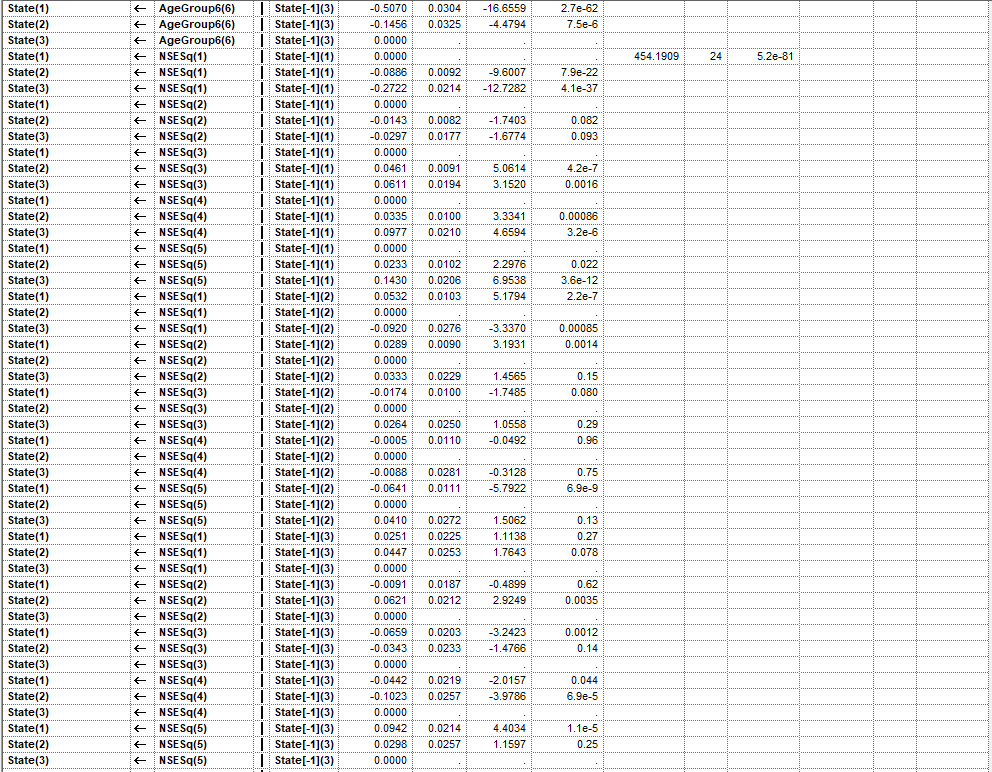


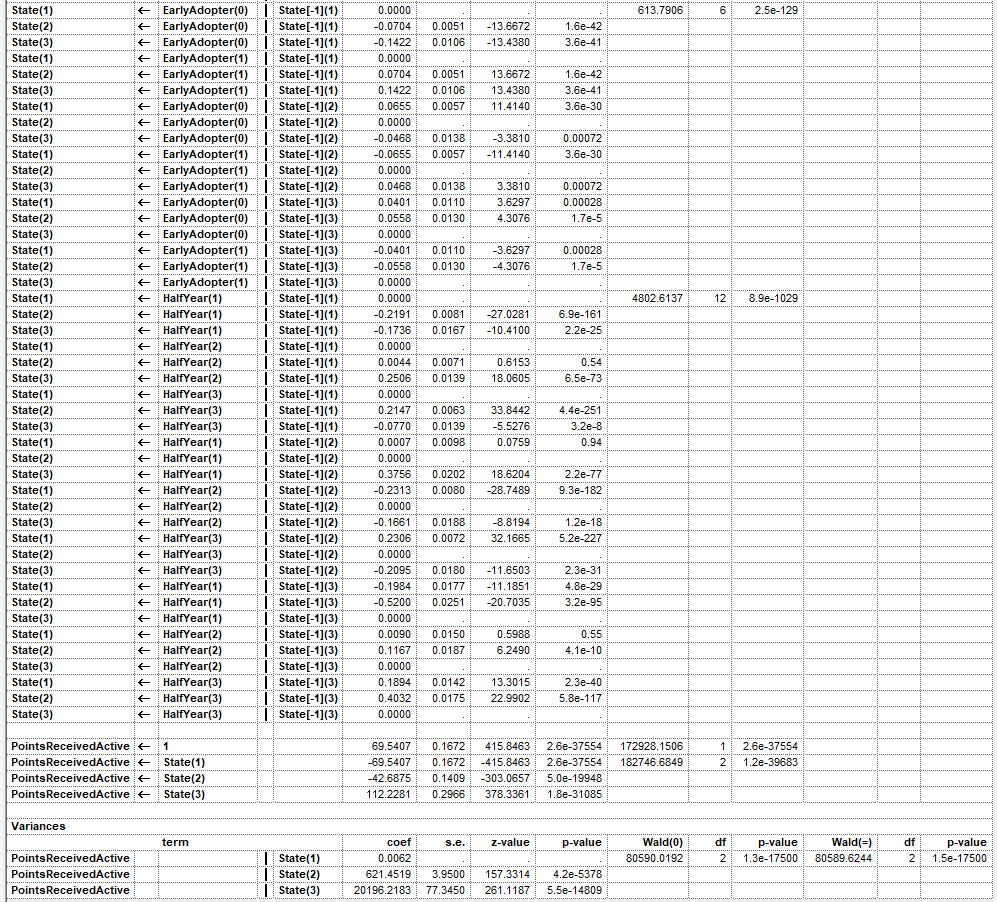


**Appendix Section 4: HMM estimation results including time dependency**

Appendix Table 5. HMM estimation results: posterior probability means accounting for different time periods

|  | 1 – Inactivity | 2 – Average activity | 3 – High activity |
| --- | --- | --- | --- |
| First half year | 0.72 | 0.14 | 0.14 |
| Second half year | 0.65 | 0.20 | 0.15 |
| Third half year | 0.69 | 0.20 | 0.11 |

Appendix Table 6. HMM estimation results: transition matrices accounting for different time periods


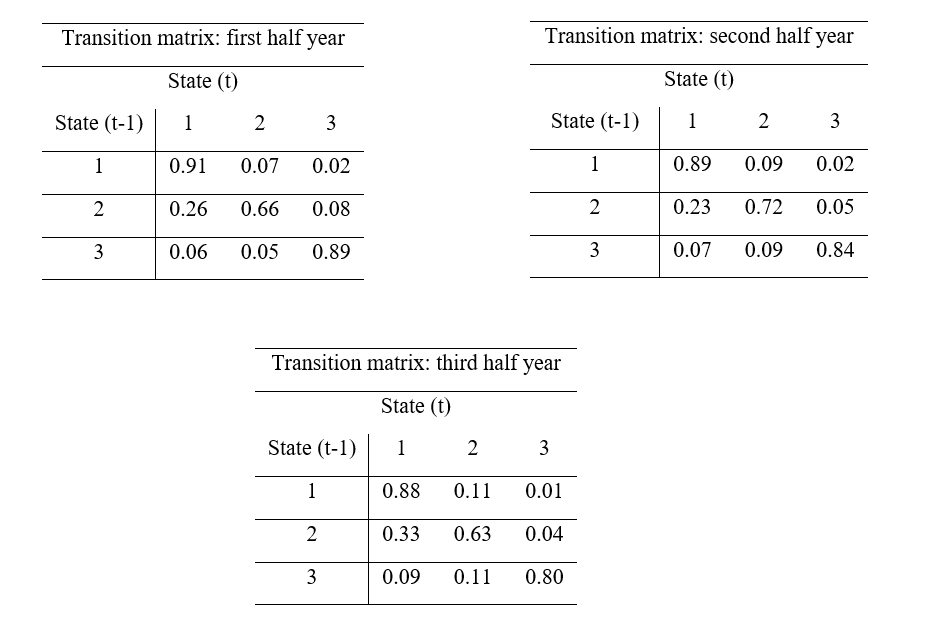


Appendix Table 7. HMM estimation results: transition matrices for the first half year


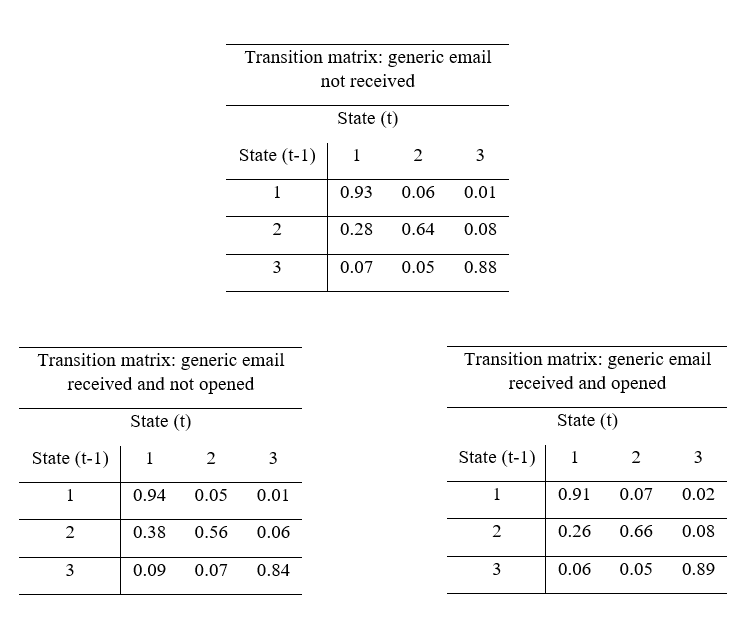


Appendix Table 8. HMM estimation results: transition matrices for the second half year


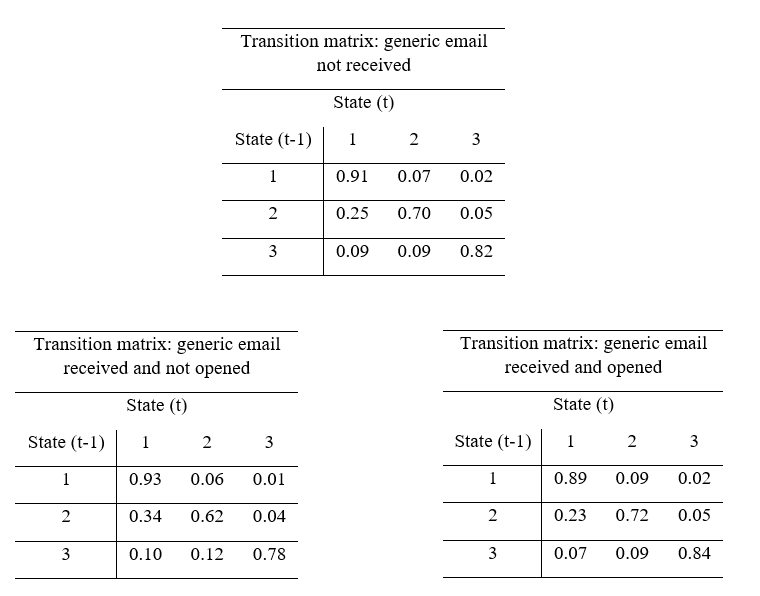


Appendix Table 9. HMM estimation results: transition matrices for the third half year


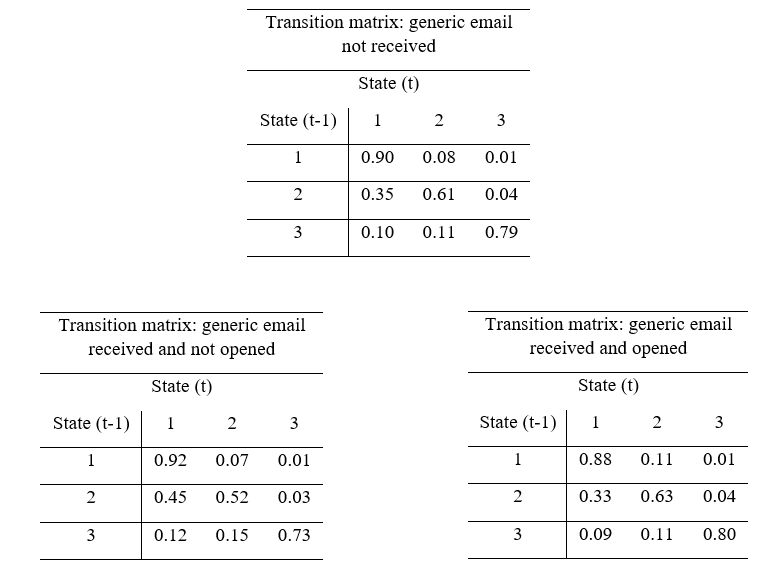


**Appendix Section 5: HMM estimation results including background characteristics**

Appendix Table 10. HMM estimation results: posterior probability means accounting for gender

|  | 1 – Inactivity | 2 – Average activity | 3 – High activity |
| --- | --- | --- | --- |
| Male | 0.65 | 0.18 | 0.17 |
| Female | 0.71 | 0.19 | 0.10 |

Appendix Table 11. HMM estimation results: transition matrices for males


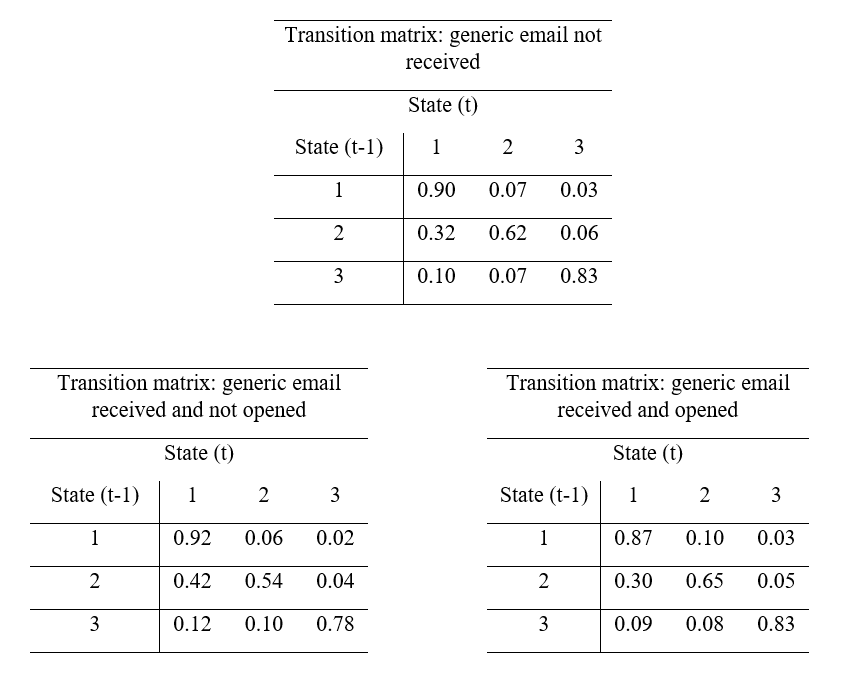


Appendix Table 12. HMM estimation results: transition matrices for females


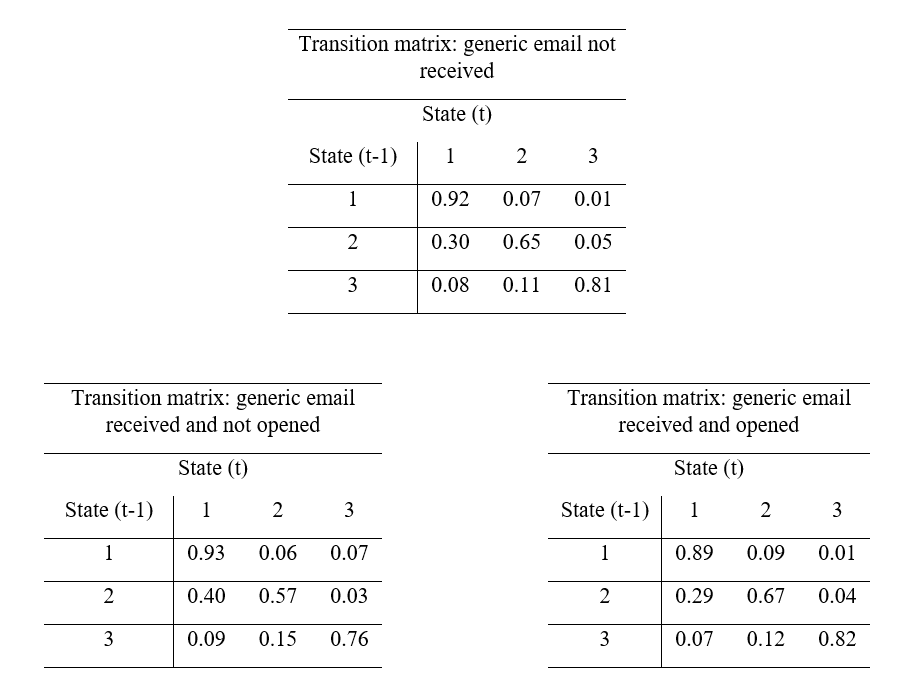


Appendix Table 13. HMM estimation results: posterior probability means accounting for NSES quintile

|  | 1 – Inactivity | 2 – Average activity | 3 – High activity |
| --- | --- | --- | --- |
| NSES_1 | 0.73 | 0.17 | 0.10 |
| NSES_2 | 0.69 | 0.18 | 0.12 |
| NSES_3 | 0.67 | 0.19 | 0.14 |
| NSES_4 | 0.67 | 0.19 | 0.14 |
| NSES_5 | 0.67 | 0.20 | 0.13 |

Appendix Table 14. HMM estimation results: transition matrices for the lowest NSES quintile


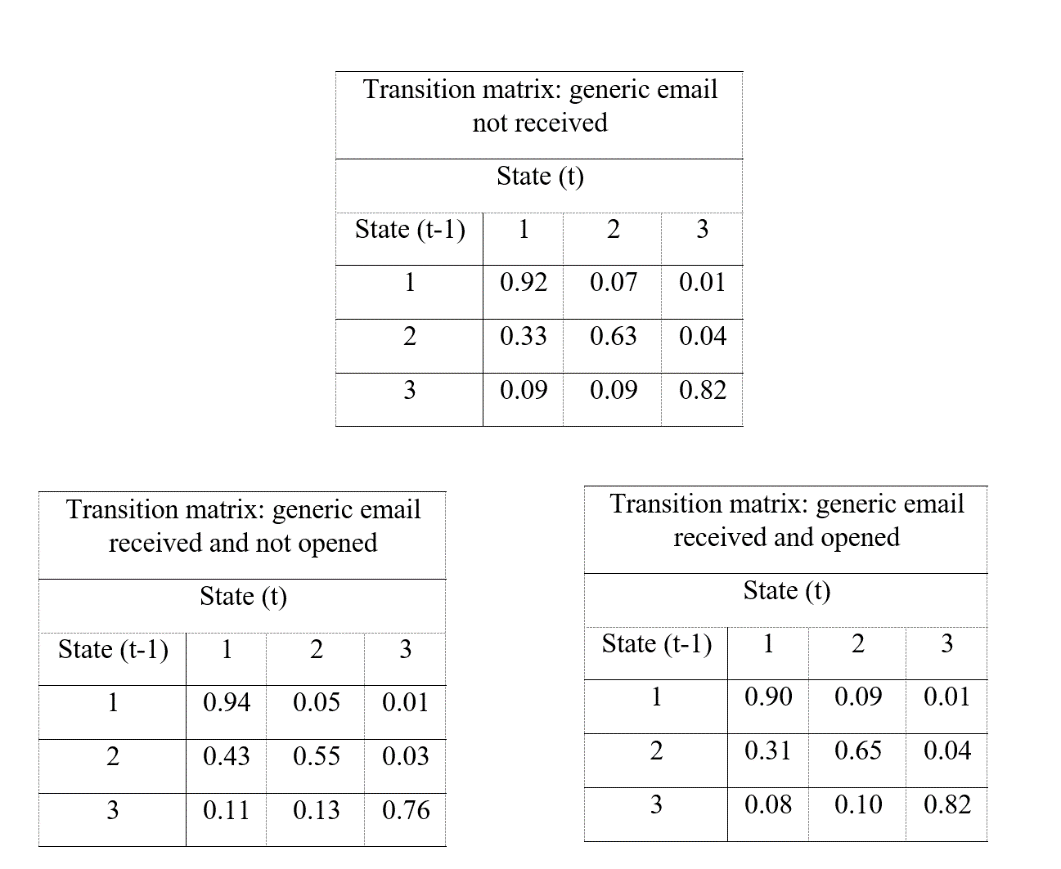


Appendix Table 15. HMM estimation results: transition matrices for the highest NSES quintile


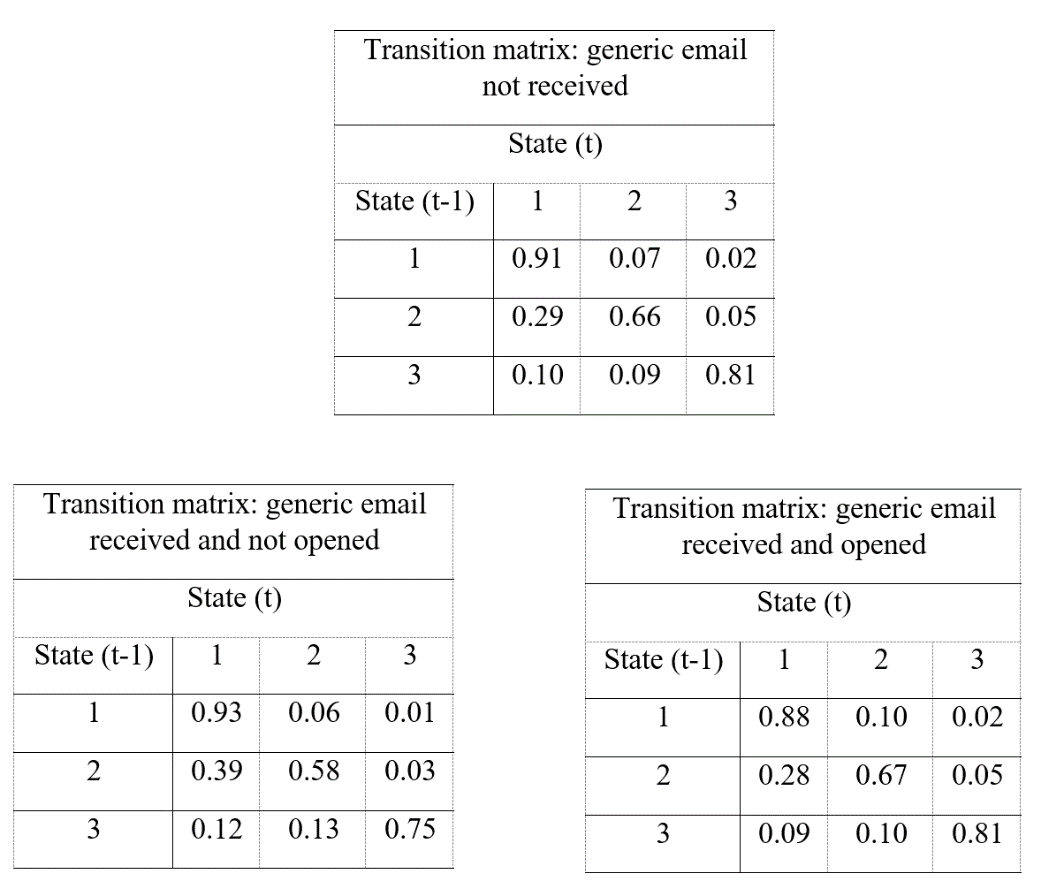


Appendix Table 16. HMM estimation results: posterior probability means accounting for age groups

|  | 1 – Inactivity | 2 – Average activity | 3 – High activity |
| --- | --- | --- | --- |
| 18-26 | 0.81 | 0.14 | 0.05 |
| 27-36 | 0.76 | 0.16 | 0.08 |
| 37-46 | 0.69 | 0.19 | 0.12 |
| 47-56 | 0.65 | 0.20 | 0.15 |
| 57-66 | 0.67 | 0.19 | 0.14 |
| 67-80 | 0.67 | 0.20 | 0.13 |

Appendix Table 17. HMM estimation results: transition matrices for the age group 18 to 26 years (1)


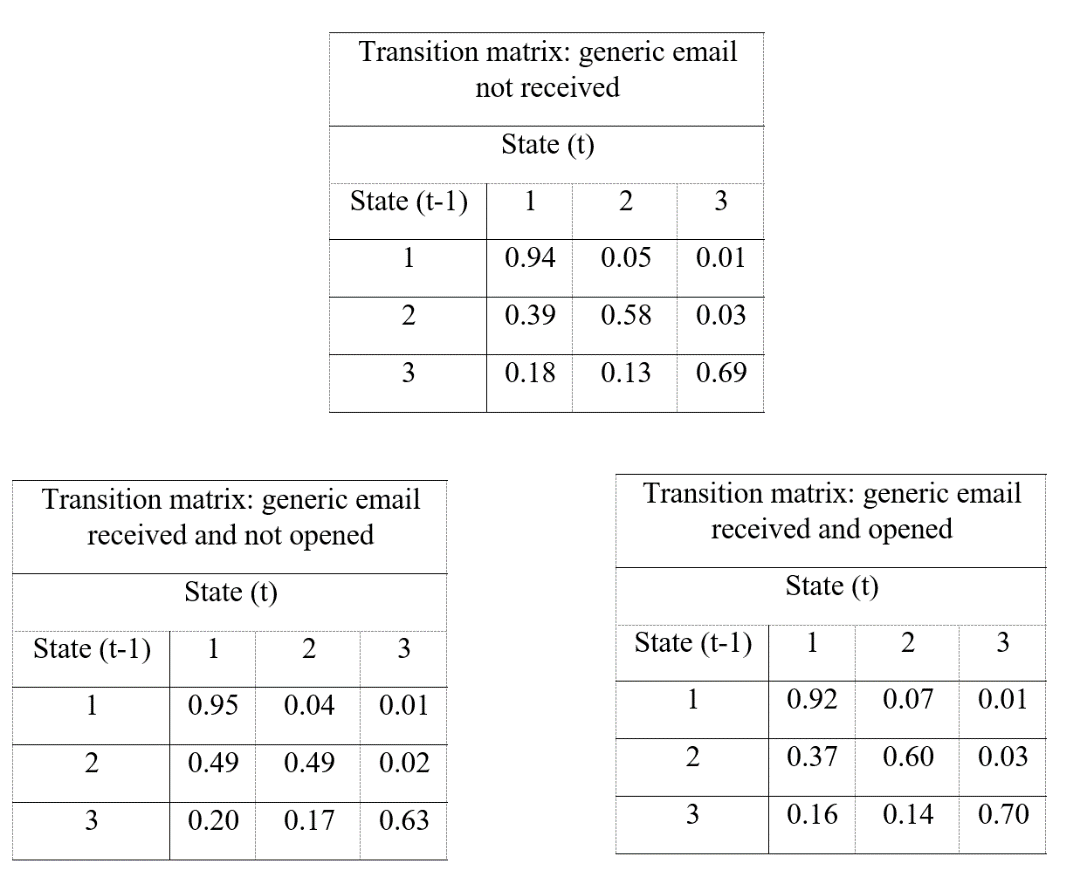


Appendix Table 18. HMM estimation results: transition matrices for the age group 47 to 56 years (4)


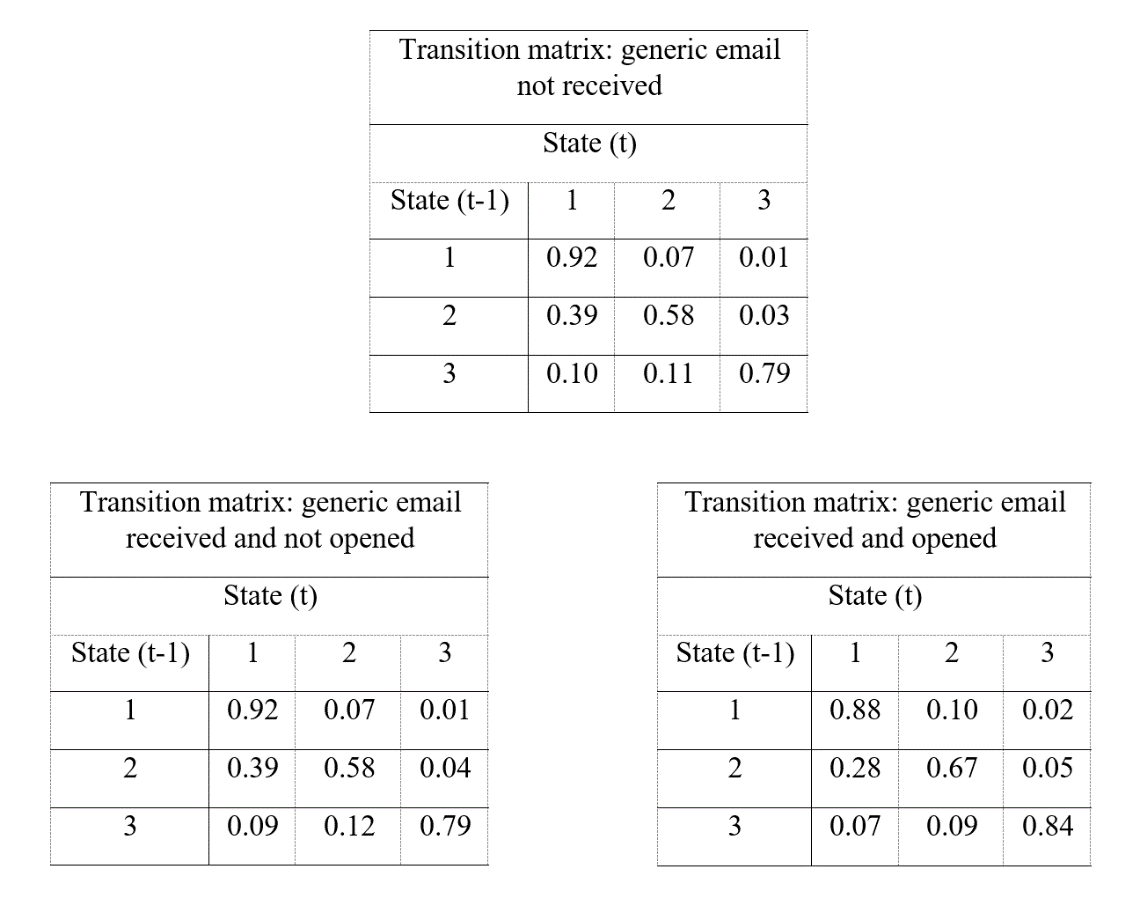


Appendix Table 19. HMM estimation results: transition matrices for the age group 67 to 80 years (6)


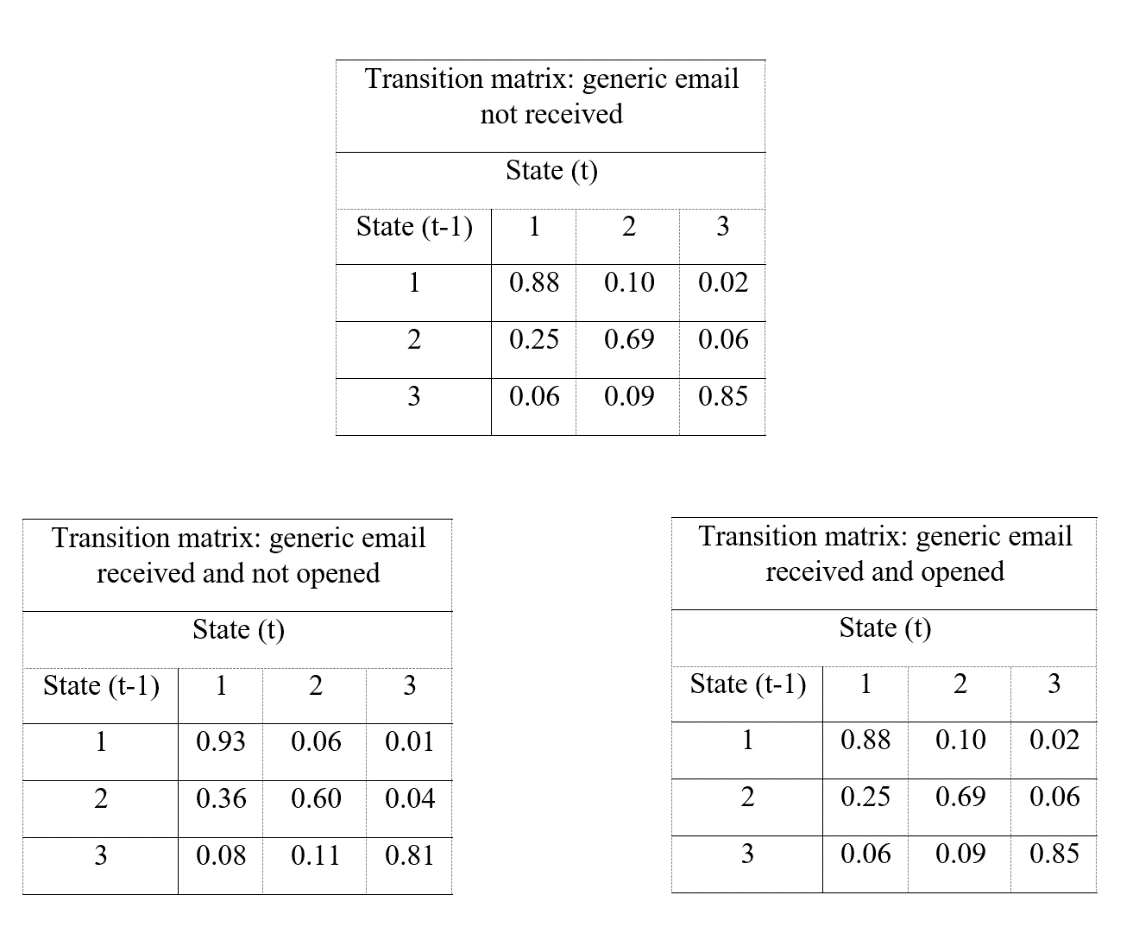


Appendix Table 20. HMM estimation results: posterior probability means accounting for early adopters

|  | 1 – Inactivity | 2 – Average activity | 3 – High activity |
| --- | --- | --- | --- |
| Early adopter | 0.66 | 0.19 | 0.15 |
| Not early adopter | 0.70 | 0.19 | 0.11 |

Appendix Table 21. HMM estimation results: transition matrices for early adopters


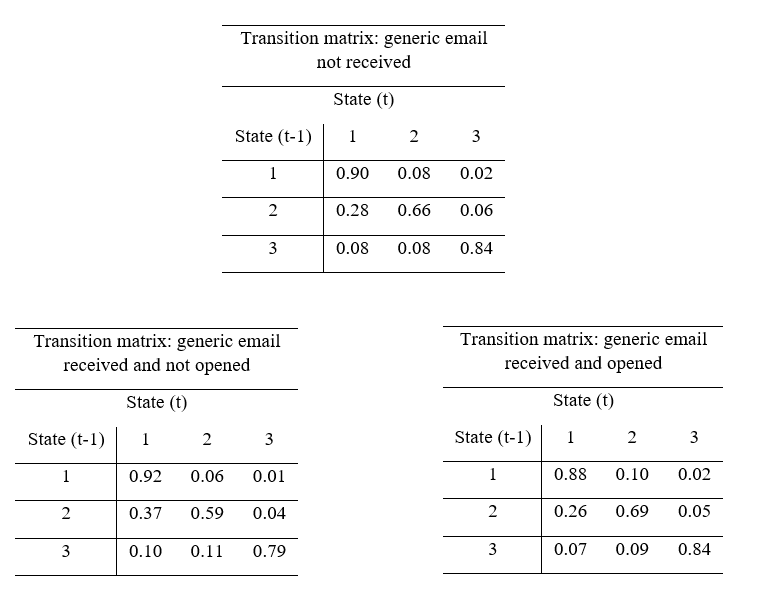


Appendix Table 22. HMM estimation results: transition matrices for not early adopter


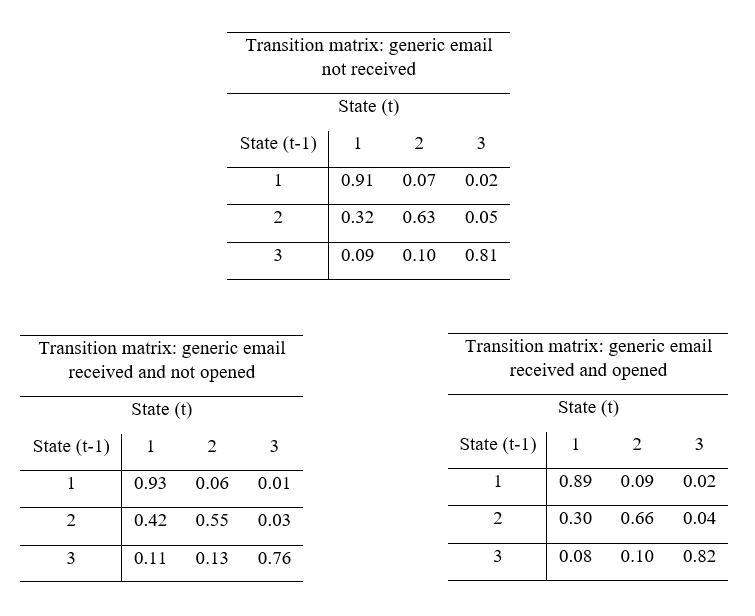


**Appendix Section 6: Alternative model specifications**

Specification 1: using as email prompt an indicator for any email received and opened (independent of email type)

Appendix Table 23. HMM estimation results: posterior probability means with indicator for any email received/opened

|  | 1 – Inactivity | 2 – Average activity | 3 – High activity |
| --- | --- | --- | --- |
| No email received | 0.68 | 0.19 | 0.13 |
| Email received and not opened | 0.79 | 0.15 | 0.06 |
| Email received and opened | 0.67 | 0.22 | 0.11 |

Appendix Table 24. HMM estimation results: transition matrices for any email


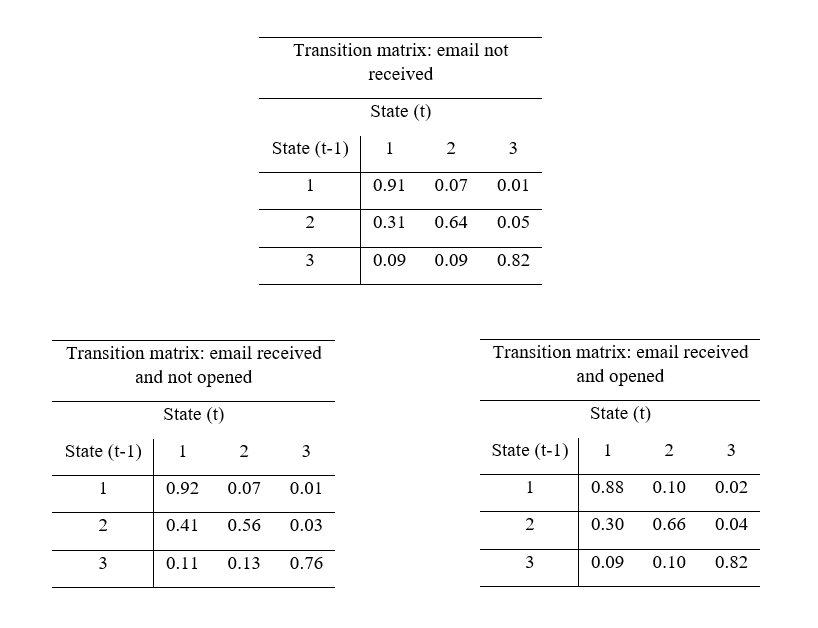


Note: Receiving and opening any email (while not differentiating between emails based on targeting nature or topic) is associated with a 3 percentage point decrease in the probability of remaining in the inactivity state, this being similar to the effect using generic emails discussed in this study.

Specification 2: adding generic emails as covariates in the response probabilities model

Appendix Table 25. HMM model fit and parameter estimates with generic emails in response probabilities


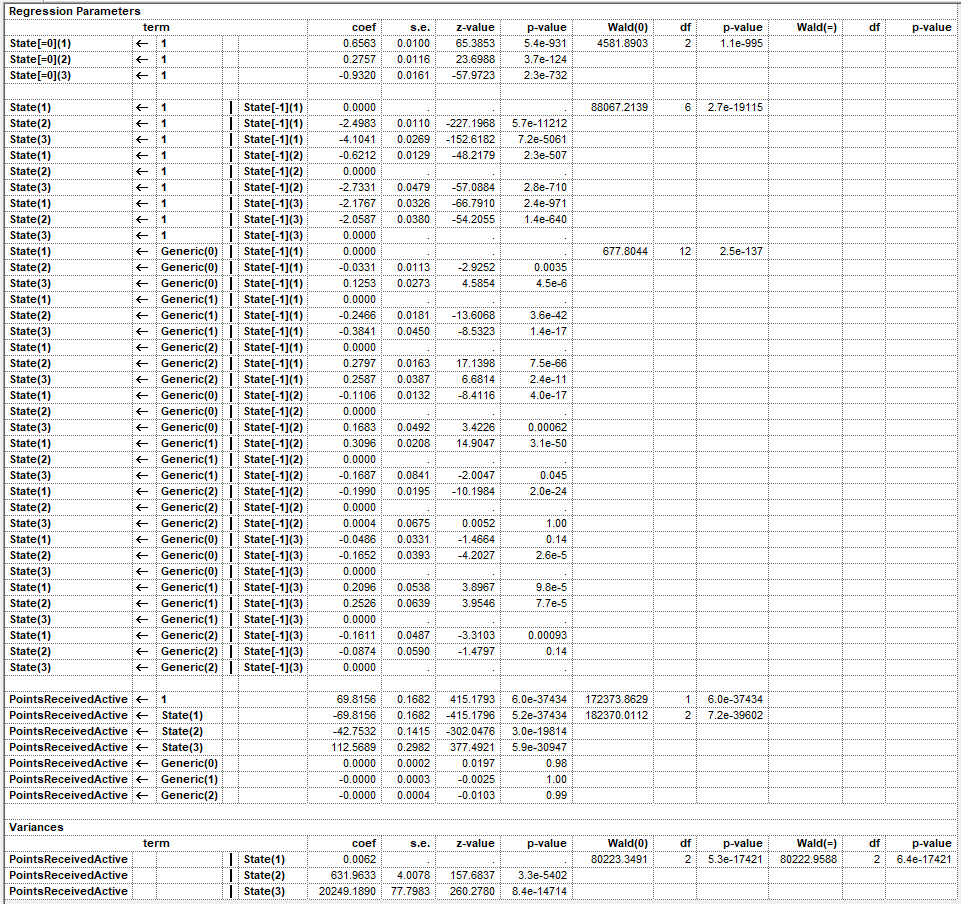


Note: The p-values for the parameter estimates associated with generic emails influencing points gained within the response probabilities model indicate a statistically insignificant relationship: p-values of 0.98, 1.00, and 0.99 for the values of not receiving a generic email (Generic-0), receiving and not opening a generic email (Generic-1), and receiving and opening a generic email (Generic-2) respectively. This implies that the generic email prompts do not have a statistically significant effect as covariates in the response probabilities model, suggesting that the main HMM analysed in the study is a more accurate representation.
